# Dental care for early childhood in Brazil: from the public policy to evidence

**DOI:** 10.11606/S1518-8787.2019053000540

**Published:** 2019-01-18

**Authors:** Gustavo Essvein, Alexandre Baumgarten, Rafaela Soares Rech, Juliana Balbinot Hilgert, Matheus Neves

**Affiliations:** I Universidade Luterana do Brasil. Curso de Odontologia. Canoas, RS, Brasil; II Universidade Federal do Rio Grande do Sul. Programa de Pós-Graduação em Epidemiologia. Porto Alegre, RS, Brasil; III Universidade Federal do Rio Grande do Sul. Programa de Pós-Graduação em Odontologia. Porto Alegre, RS, Brasil

**Keywords:** Child, Preschool, Dental Care for Children, Comprehensive Health Care, Dental Health Services, Public Health Dentistry, Pré-Escolar, Assistência Odontológica para Crianças, Assistência Integral à Saúde, Serviços de Saúde Bucal, Odontologia em Saúde Pública

## Abstract

**OBJECTIVE:**

To evaluate whether characteristics of health services, oral health team and dental surgeon are associated with provision of dental care for children up to five years old in Brazilian Primary Health Care.

**METHODS:**

A cross-sectional study was conducted with data from 18,114 oral health teams in Brazil, evaluated in 2014 by the National Program for Access and Quality Improvement in Primary Care. The study outcome was the proven performance of dental procedures on children up to five years old. Statistical analysis was performed by Poisson regression based on a hierarchical model, where the first level was composed of service organization variables, the intermediate level composed of unit planning characteristics, and the proximal level composed of variables related to dental surgeon characteristics.

**RESULTS:**

Prevalence of dental care performed by oral health teams was 80.9% (n = 14,239). Scheduled appointments and activities of education in health were positively associated with the outcome, as well as planning and programming activities for the population and monitoring and analysis of oral health indicators. Complementary training in public health, continuing education activities and career plan were variables related to dental surgeons associated with the service provision.

**CONCLUSIONS:**

One fifth of health units in Brazil do not provide dental care for children in early childhood. Health units’ well-structured organization and planning protocols are associated with the provision of this service, as well as better employment relationship and graduate activities for dental surgeons.

## RESUMO

**OBJETIVO:**

Avaliar se características dos serviços de saúde, da equipe de saúde bucal e do cirurgião-dentista estão associadas à prestação de atendimento odontológico a crianças de até cinco anos de idade na atenção básica brasileira.

**MÉTODOS:**

Estudo transversal, com dados de 18.114 equipes de saúde bucal do Brasil avaliadas pelo Programa Nacional de Melhoria do Acesso e da Qualidade em 2014. O desfecho do estudo foi a realização comprovada de procedimentos odontológicos em crianças de até cinco anos de idade. A análise estatística foi feita por regressão de Poisson com base em um modelo hierárquico, sendo o primeiro nível composto por variáveis de organização do serviço, o nível intermediário por características de planejamento da unidade e o nível proximal por variáveis relacionadas ao cirurgião-dentista.

**RESULTADOS:**

A prevalência de realização do atendimento odontológico pelas equipes de saúde bucal foi de 80,9% (n = 14.239). Consultas agendadas e atividades de educação em saúde se associaram positivamente ao desfecho, bem como atividades de planejamento e programação para a população e o monitoramento e análise de indicadores de saúde bucal. Formação complementar em saúde pública, atividades de educação permanente e plano de carreira foram variáveis relacionadas aos cirurgiões-dentistas que se associaram à prestação do serviço.

**CONCLUSÕES:**

Um quinto das unidades de saúde do Brasil não realiza atendimento odontológico na primeira infância. Protocolos de organização e planejamento bem-estruturados nas unidades de saúde estão associados à realização desse atendimento, bem como melhores vínculos trabalhistas e atividades de pós-graduação para os cirurgiões-dentistas.

## INTRODUCTION

Dental cavity is considered a serious public health issue worldwide, ant it is one of the most prevalent chronic diseases^1^. According to the latest evaluation of Brazilian population's oral health conditions, its prevalence in 5-year-old children reaches approximately 53.4%. With regard to the permanent dentition, the situation is worse, since some 70% 12-year-old children present at least one decayed tooth^2^. Poor oral health conditions may have a negative impact on overall health during early childhood, causing biopsychosocial, growth and physical development damages, in addition to decreased quality of life and learning capacity^3^ ^,^ ^4^.

Early childhood care is an achievement of dentistry and represents a new understanding of the approach to oral diseases, strongly centered on a preventive-promotional perspective. Cavities develop from multiple and complex interactions involving biological, behavioral, and social issues. The literature describes more than 100 risk factors for the development of childhood cavities^5^ ^,^ ^6^, which can be broadly categorized into sociodemographic, behavioral, dietary, related to health services, or referring to knowledge, cognition and beliefs. Most of them are modifiable by access to quality dental care in primary health care. Only 22.1% children under the age of five have been to the dentist at least once in their lifetime^7^. Resoluteness, in turn, varies according to the oral health team modality^8^.

Within this perspective, the National Oral Health Policy^9^ becomes a reference in health recovery and injury prevention to stop the progression and prevent eventual disabilities and damages coming from early childhood cavities. Dentistry changed its perspective of performing curative procedures to look at the health-disease process. This change put into practice strategies aimed at population early care, seeking to minimize and avoid sequelae of the main oral diseases. Therefore, dental care for children becomes a fundamental strategy for the reduction of sequelae at more advanced ages^10^.

Due to the epidemiological condition and need of care provision for children up to five years old in Brazil, it is necessary to know the characteristics of primary health care services and oral health teams, which may be associated with the performance of this service in the country. This study aimed at evaluating the rate of dental care provided for children up to five years old by oral health teams of the Primary Health Care, relating it to health service factors and dental surgeon characteristics.

## METHODS

A cross-sectional study was performed from data collected in a multicentric way between March and December 2014 in 24,055 primary health care units (PHCU) throughout the country. For this study, we used data from 18,114 oral health teams (OHT) evaluated in the second cycle of the National Program for Access and Quality Improvement in Primary Care (PMAQ-AB), corresponding to 81.5% of the 22,213 existing OHT.

The PMAQ-AB aims to encourage managers and teams to improve the quality of health services offered to Brazilian citizens. For this purpose, it proposes a set of strategies for qualification, monitoring and evaluation of health teams’ work. It is a national program that is implemented in multicentric way and integrated by several teaching and research institutions, besides being directly supervised by the Ministry of Health. The data from this research come from the external evaluation phase on health teams. The results found refer to the evaluations of Module VI, completed from interviews with health units’ workers and *in loco* documentary verification. The teams participating in PMAQ were able to voluntarily join the program by an initial pact between teams and municipal managers. The municipal managers completed an electronic form made available on the program website, and only the teams that formalized the membership in this way were evaluated. The data were collected in health units using tablets, which presented an app with a standardized and previously tested instrument. After the external evaluation, the data collected were sent via the Internet to a Ministry of Health server for validation.

The selected external evaluators participated in training with the same field manual. A data collection qualification protocol was established, and it consisted of five criteria: geographic coordinates captured by the instrument, data collection time per module, evaluation start and end times, percentage of unanswered alternatives, and responses with repeated characters.

The study outcome was the proven performance of dental procedures on children up to five years old, evaluated by the questions: “Does the oral health team provide service for children up to five years old?” “Is there any document to prove it?” For documentary evidence, child's monitoring record in the odontogram and e-SUS records were considered. Care provision outcome was considered positive when both responses were positive.

Independent variables were collected related to OHT organization and planning actions and to dental surgeon characteristics. In the OHT organization group, the variables related to the oral health team modality were included as follows: type I, comprising one dental surgeon and one oral health assistant (ASB); type II, comprising one dental surgeon, one ASB and one oral health technician (TSB); and parameterized OHT, which comprises two dental surgeons, each one with a workload of 20 hours/week, one ASB and one TSB. Regarding clinical service shifts, the teams were classified into OHT with one work shift and OHT with two or more work shifts. Answers to the question “On which days of the week does the oral health team work in this unit?” were collected in each work shift independently and dichotomized between one to four days of work/week or five to seven days of work/week. For the question “Is the oral health team's schedule shared with family health team professionals?”, the possible answers were ‘yes’ or ‘no’. Finally, the appointments scheduling was classified into: all scheduled; most scheduled and others by spontaneous demand; half scheduled and half by spontaneous demand; none scheduled and all by spontaneous demand.

Among the variables of the OHT planning actions group, the following variables were used: planning and programming performed by the oral health team, performed by the primary health care team (EAB), or not performed; monitoring and analysis of oral health indicators or not; support for OHT planning or not; presence or lack of information to support health analysis; planning of activities by OHT according to local information or not.

Finally, the variables referring to the dental surgeon characteristics were grouped, namely: complementary training or not; Consolidation of Labor Laws (CLT), statutory, temporary or other modalities of employment relationship (subdivided into commissioned position, autonomous, other, do not know or did not answer); presence or lack of a career plan; number of continuing education activities performed (between zero and three, four and six, or more than seven activities), including seminars, face-to-face courses, Telemedicine University Network (RUTE) courses, Open University of the Unified Health System (UNA-SUS), distance education courses, exchange of experience, tutoring or pedagogical support, teaching in service, and other modalities.

Statistical analysis was performed with Stata 12.0 software. The association between the predictors and the outcome was assessed by descriptive and bivariate analyzes using the chi-square test (p < 0.05). The multivariate analysis was performed by Poisson regression with robust variance, resulting in the prevalence ratio (PR) and confidence interval (95%CI). A hierarchical model was elaborated for the analysis (Figure), where the first level was composed of the service organization variables (oral health team modality, service shifts, work days, schedule sharing, education activities and scheduled appointments), the intermediate level composed of the variables related to the activities of the health units (planning and programming activities, monitoring and analysis of oral health indicators, support for planning, availability of information to assist health analysis and planning of activities by OHT according to local information), and the proximal level composed of variables related to the dental surgeon (complementary training, employment relationship, career plan and number of education activities). The variables were selected by the stepwise backward procedure. Exposure variables with p < 0.20 were included in the multivariate analysis. The final model included the variables associated with the outcome (p < 0.05).

**Figure 1:**
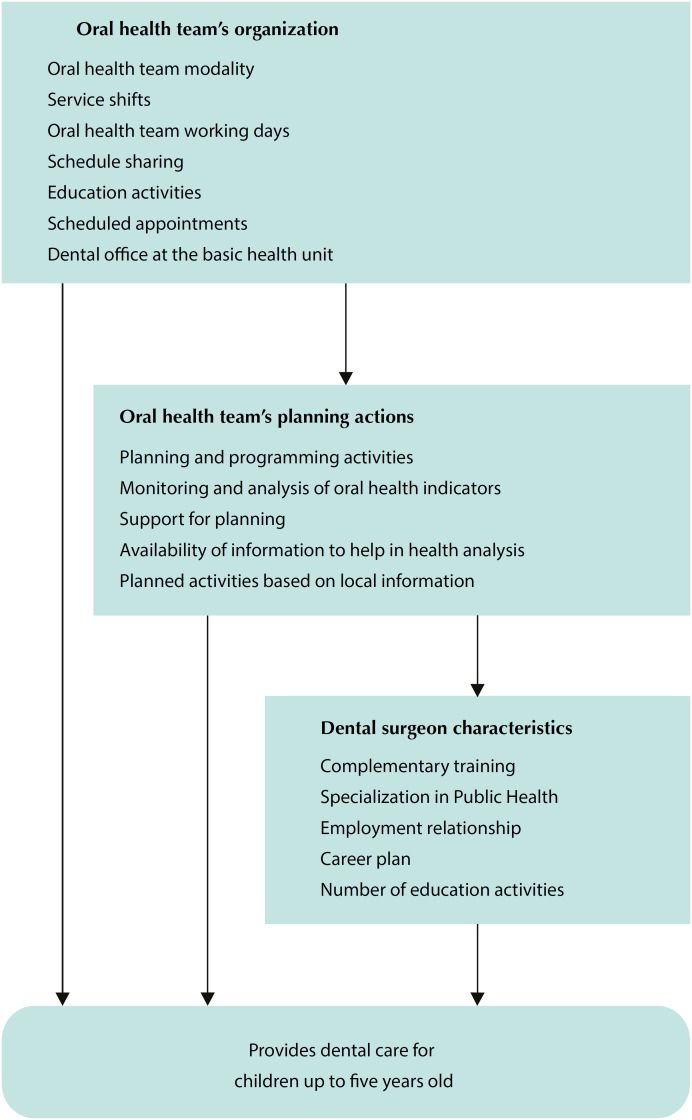
Hierarchical model of analysis.

This study was approved by the Committee of Ethics in Research with Human Beings of the Universidade Federal do Rio Grande do Sul, Protocol 21,904, and meets relevant bioethical legislation.

## RESULTS

Among the 18,114 OHT evaluated, 2.9% (n = 530) did not provide dental care, and 18.5% (n = 3,345) did not present service supporting documentation. The prevalence of dental care for children up to five years old at the OHT was 80.9% (n = 14,239). Due to the lack of data on at least one variable, 4,014 OHT were not included in the final multiple regression model. Regarding organization variables, 86.3% OHTs were type I; the oral health schedule was shared (67.0%) in most PHCU; 92.0% OHT worked in two shifts or more, and 74.0% appointments were previously scheduled. With regard to planning actions, 79.0% teams received support, and 80.0% of them planned activities according to local information. As for the dental surgeon characteristics, only 33.0% professionals are specialized in family health; 44.0% of them have statutory employment relationship, and most of them (72.0%) participate in zero to three education activities (Tables 1 and 2).

**Table 1:** Prevalence of care provision for children up to five years old by the oral health team (OHT), according to independent variables (organization and service actions). Brazil, 2014.

Variable		n	Provide oral health care		p^a^
			n = 14,239	%	
Service organization					
OHT modality^b^					< 0.001
	1	13,982	10,787	79.6	
	2	1,928	1,592	84.6	
Parameterized		292	220	79.1
Service shifts^b^					< 0.001
	1	1,306	904	73.0	
	≥ 2	14,896	11,695	81.0	
OHT work days/week^b^					0.028
	1–4	15,184	11,847	80.4	
	5–7	1,018	752	77.5	
Schedule sharing					< 0.001
	Yes	12,007	9,734	82.4	
	No	6,107	4,505	77.2	
Education activities					< 0.001
	Yes	16,407	13,175	82.1	
	No	1,707	1,064	69.0	
Scheduled appointments					< 0.001
	Only spontaneous demand	796	466	64.0	
	Half scheduled and half by spontaneous demand	3,054	2,405	81.0	
	Most scheduled	13,373	10,690	82.0	
	Only scheduled	891	678	79.3	
Dental office at the PHCU					< 0.001
	Yes	14,835	11,662	81.0	
	No	1,367	937	71.8	
Actions					
Planning and programming activities					< 0.001
	Yes, with oral health team	3,504	2,671	78.6	
	Yes, with primary health care team	11,446	9,406	83.7	
	No	3,164	2,162	73.4	
Monitoring and analysis of oral health indicators					< 0.001
	Yes	12,031	9,940	84.3	
	No	6,083	4,299	74.3	
Support for planning					< 0.001
	Yes	14,303	11,605	83.0	
	No	3,811	2,634	73.1	
Availability of information to assist in health analysis					< 0.001
	Yes	13,304	10,856	83.4	
	No	4,810	3,383	74.0	
Planned activities based on local information					< 0.001
	Yes	14,518	11,763	82.8	
	No	3,596	2,476	73.4	

PHCU: Primary Health Care Unit

a Chi-square test (p < 0.05).

b Lack of data.

**Table 2:** Prevalence of care provision for children up to five years old by the oral health team (OHT), according to independent variables (dental surgeon). Brazil, 2014.

Variable		n	Provide oral health care		p^a^
			n = 14,239	%	
Dental surgeon					
Complementary training					< 0.001
	Yes	12,581	10,225	83.1	
	No	5,533	4,014	76.0	
Specialization in family health care					< 0.001
	Yes	4,128	3,472	85.6	
	No	8,453	6,753	82.0	
Employment relationship					< 0.001
	CLT	2,997	2,438	83.4	
	Statutory	7,993	6,239	81.0	
	Others	753	538	78.5	
	Temporary	6,396	5,024	80.3	
Career plan^b^					< 0.001
	Yes	3,640	3,005	85.0	
	No	13,943	10,878	80.2	
Number of education activities^b^					< 0.001
	0–3	13,020	9,831	78.3	
	4–6	4,532	3,894	87.1	
	≥ 7	587	514	91.9	

CLT: Consolidation of Labor Laws

a Chi-square test (p < 0.05).

b Lack of data.

Regarding OHT variables, the highest prevalence of the outcome was found in OHT type II (p < 0.001), with the presence of a dental office at the PHCU (p < 0.001), with two or more service shifts (p < 0.001), in teams that share schedules (p < 0.001), with education activities (p < 0.001) and scheduled appointments (p < 0.001) (Table 1).

Most teams that provided care for children up to five years old planned programming along with the EAB (p < 0.001), monitored and analyzed oral health indicators (p < 0.001), received support for planning (p < 0.001), had information to help in health analyzes (p < 0.001), and planned activities according to local information (p < 0.001) (Table 1).

Regarding the dental surgeon characteristics, complementary training (p < 0.001), specialization in family health (p < 0.001), CLT or statutory employment relationship (p < 0.001), career plan (p < 0.001) and higher number of educational activities (p <0.001) were positively associated with provision of dental care for children (Table 2).

In the multivariate analysis, after adjustment to potential confusing elements for the OHT organization variables, the prevalence of the outcome was significantly higher (PR = 1.05, 95%CI 1.03–1.08) for OHT type II; 13% higher (PR = 1.13, 95%CI 1.08–1.19) for those which had two or more service shifts; 24% higher (PR = 1.24, 95%CI, 1.17–1.31) when most users were scheduled, and 20% higher (PR = 1.20, 95%CI 1.17–1.34) when the OHT schedule included education activities. Regarding the OHT planning activities, the probability of outcome after adjustment was 7% higher (PR = 1.07, 95%CI 1.04–1.06) when the EAB performed planning and programming activities, and 6% higher (PR = 1.06, 95%CI 1.04–1.08) when monitored and analyzed oral health indicators (Table 3). Regarding the dental surgeon characteristics, the complementary training was related to an 8% greater outcome probability (PR = 1.08, 95%CI 1.03–1.10), and seven or more continuing education activities related to a 6% greater probability (PR = 1.06, 95%CI 1.02–1.09) (Table 4).

**Table 3:** Crude and adjusted prevalence ratios of the outcome “dental appointment up to five years old” according to independent variables (service organization and actions).

Variable		Crude			Adjusted		
		PR	95%CI	p	PR	95%CI	p
Service organization							
OHT modality				< 0.001			< 0.001
	1	1.00			1.00		
	2	1.06	1.04–1.09		1.05	1.03–1.08	
	Parameterized	0.99	0.93–1.06		1.00	0.94–1.06	
Service shifts				< 0.001			< 0.001
	1	1.00			1.00		
	≥ 2	1.11	1.07–1.15		1.13	1.08–1.19	
OHT work days/week				0.049			0.005
	1–4	1.00			1.00		
	5–7	0.96	0.93–1.00		1.07	1.02–1.12	
Schedule sharing				< 0.001			< 0.001
	Yes	1.07	1.06-1.09		1.05	1.03–1.07	
	No	1.00			1.00		
Education activities				< 0.001			< 0.001
	Yes	1.39	1.22–1.57		1.20	1.17–1.34	
	No	1.00			1.00		
Scheduled appointments				< 0.001			< 0.001
	Only spontaneous demand	1.00			1.00		
	Half scheduled and half by spontaneous demand	1.27	1.19–1.34		1.23	1.15–1.30	
	Most scheduled	1.28	1.21–1.35		1.24	1.17–1.31	
	Only scheduled	1.24	1.16–1.32		1.21	1.13–1.29	
Actions							
Planning and programming activities				< 0.001			< 0.001
	Yes, with oral health team	1.07	1.04-1.10		1.03	0.99-1.06	
	Yes, with primary health care team	1.14	1.11-1.17		1.07	1.04-1.10	
	No	1.00			1.00		
Monitoring and analysis of oral health indicators				< 0.001			< 0.001
	Yes	1.13	1.11–1.15		1.06	1.04–1.08	
	No	1.00			1.00		
Support for planning				< 0.001			0.005
	Yes	1.13	1.10–1.16		1.04	1.01–1.07	
	No	1.00			1.00		
Availability of information to help in health analysis				< 0.001			0.028
	Yes	1.13	1.11–1.15		1.03	1.01–1.05	
	No	1.00			1.00		
Planned activities based on local information				< 0.001			0.034
	Yes	1.13	1.10–1.15		1.03	1.01–1.05	
	No	1.00			1.00		

OHT: Oral Health Team

**Table 4:** Crude and adjusted prevalence ratios of the outcome “dental appointment up to five years old” according to independent variables (dental surgeon).

Variable		Crude			Adjusted		
		PR	95%CI	p	PR	95%CI	p
Dental surgeon							
Complementary training				< 0.001			< 0.001
	Yes	1.09	1.08–1.11		1.08	1.03–1.10	
	No	1.00			1.00		
Employment relationship				< 0.001			< 0.001
	CLT	1.0			1.0		
	Statutory	0.97	0.95–0.99		0.97	0.95–0.99	
	Others	0.94	0.90–0.98		0.97	0.92–1.02	
	Temporary	0.96	0.94–0.98		0.98	0.96–1.00	
Career plan				< 0.001			0.020
	Yes	1.06	1.04–1.08		1.03	1.01–1.06	
	No	1.00			1.00		
Number of education activities				< 0.001			< 0.001
	0–3	1.00			1.00		
	4–6	1.11	1.10–1.13		1.04	1.02–1.06	
	≥ 7	1.17	1.14–1.21		1.06	1.02–1.09	

CLT: Consolidation of Labor Laws

## DISCUSSION

This study showed that almost a fifth of the OHT do not provide dental care for children up to five years old, recommended in Brazilian primary health care. Since the unit of analysis is the oral health team, responsible for a large population in a pre-established territory, these data become alarming since there are thousands of children without access to the dentist.

Dental care provision was associated with the teams’ work organization, with planning activities and with the dental surgeon characteristics. Although the results of the regressions are apparently of small magnitude, considering the size of the sample, they presented restricted confidence intervals, making the power of the study to detect numerically small differences visible, but with great impact on public health. Implementing changes based on scientific knowledge can support changes in country's health actions, by acts linked to strategic evidence, and which guide management. Allying research and evidence-based public health management practices enables advances in health care and major changes in communities’ oral health.

The data from this study show that, in healthcare units where the schedule is shared among professionals, there is greater probability of dental care provision. The work process of the primary health care team should consider local particularities, users’ interests, specific health issues and each community's habits and customs, as well as the peculiarities of the team itself, and the way they work. In addition, the scheduled shared between the different professionals brings to the team's knowledge specific problems of certain users.

Regarding appointments scheduling, it was verified that children up to five years old are more likely to be present and attended whit the planned scheduling. Scheduled appointments easiness may be related to preventive treatments, recognition of the dental office, demystification of the figure of the dental surgeon connected to pain, and performance of education and/or preventive appointments in a playful way, facilitating child's cooperation in future care. The search for immediate appointment, only for spontaneous demands or for curative treatments, involving pain, can often solve the immediate patient's problem, but it does not follow the longitudinal oral care relationship, fundamental for health. Schedule organization becomes important in the logistics of the team routine, raising awareness of the complexity of each case^12^.

Positive results for the workload of 40 hours/week in the primary health care team are based on the creation of a relationship between the professionals and the population. This high workload allows social and corporate work processes to develop in a context of interaction and communication. Thus, it is also possible to reserve specific times for team meetings and specific case discussions. The dental surgeon, as a member of the oral health team, should be an active participant in community actions, making part of his schedule available for several activities in addition to the office service, such as: meetings with the health team and population, planning and evaluation of ongoing individual and collective actions, home visits and deep knowledge of the population and its living conditions^13^ ^,^ ^14^.

Planning and organizing health actions does not only refer to the everyday actions, such as health care or administrative ones. The emphasis is on the possibility of constructing group processes, of a collective desire project, aiming at encouraging team members’ proactivity in a functional vision, understanding that the capacity for decision making is fundamental^11^ ^,^ ^15^.

Monitoring and analysis of oral health indicators are essential for observing health inequities. This study showed that monitoring is related to dental care planning and provision. Seeking equity in providing care and promoting population's health, knowing these indicators allows understanding the associated social conditions, besides changing the current situation of inequalities in dental services access and use^16^ ^,^ ^17^.

All these actions and planning are aimed at improving the quality and management of patient care, providing not only the correct diagnosis and treatment, but also directing them to disease prevention. When we speak of prevention, we should know that it is paramount in the health of the entire population, but especially in child's health, since it is at this age that harmful habits are often modified, and primary care is the ideal field for this. Oral health, within the scope of primary care and health surveillance, is confronted with frequent daily difficulties, and it is always necessary to find effective solutions to solve these challenges. Special attention should be given to early childhood care, since cavities attacked an average of 2.8 teeth in Brazilian children up to five years old in 2003^18^. In 2010, the mean was 2.43 affected teeth^19^, representing a reduction of 13.9% in seven years – epidemiological data that still need to be improved. There is need to improve strategies and actions for access and promotion of oral health in primary care, seeking to create a culture of providing preventive care over curative care.

The data presented in this study show that professionals with complementary training, such as specialization in collective health or public health, master's degree and doctorate, provide more dental care to children. The basic curriculum of undergraduate dentistry in Brazil has been the subject of reformulations aiming at the change of the egress health professional, seeking to meet SUS needs. It is necessary that this professional presents skills and abilities built from his basic training, which should be in constant improvement^20^.

The career plan, along with other subsidies by strategic management, becomes a key strategic tool. A study performed in Rio de Janeiro identified that the absence of a career plan was one of the factors associated with the lack of interest and turnover of professionals working in the Family Health Strategy^21^. The most prevalent type of employment relationship (42.1%) in all population strata is statutory^22^.

Work process training consists of offer of continuing education courses to primary care professionals, constituting a powerful work management tool, which contributes to worker's valorization and satisfaction. Studies report that the greater the size of the municipality, the greater the percentage of continuing education actions. It is also known that previous training and offer of continuing education are the main factors for lower professional turnover, since they help consolidate and amplify the access of primary care professionals to qualification^21^ ^,^ ^22^.

Despite the relevance of this national study, limitations deserve to be discussed. The outcome was assessed by self-reported responses on dental care provision; however, this limitation was mitigated by documentary evidence. Moreover, contextual and individual variables were evaluated via multivariate analysis, as well as their influence on the outcome. Ideally, the multilevel analysis would be better to measure this relationship, since it allows evaluating how individuals can be influenced by their social context, besides being based on the perception that the distribution of health and disease in the population cannot be only explained by individual characteristics^23^. Therefore, the conclusions of the findings should be interpreted with caution, since large samples may present an excess statistical power with significant variables, but reduced effect. However, large samples have the power to detect small differences.

Regarding the potential of this study, this is a first national study that evaluated the prevalence of dental appointments for children up to five years old from an epidemiological perspective, considering the number of teams evaluated in primary health care (ABS) and using multivariate analysis. In addition, the data come from a program of the Ministry of Health that presents a systematic evaluation for services provision according to population's real and concrete needs (greater resoluteness). It is also possible to mention the considerable size of the sample, which gives high power to the study. In addition, more than 80% oral health teams from different regions of the country participated in the research, contributing to the generation of a more complete ABS scenario.

The importance of early dental appointment up to five years old is that the family (unit) receives guidelines for oral health promotion that may prevent adverse effects of recognized negative impact on the quality of life of children and their relatives^24^ ^,^ ^25^. In addition, studies show that investing in the first oral health care causes lower costs in dental treatment^10^.

The study showed the influence of work organization, OHT planning and dental surgeon characteristics on the prevalence of oral appointments for children up to five years old. The results demonstrate management ways to be followed, being the basis for the implementation of public health policies focused on the qualification of the service provided and the incentive to early pediatric dental care.

Thus, it is concluded that early childhood care was more frequent in services with OHT type II, dental office at the PHCU, two or more care provision shifts, and schedule with specific educational activities shared with other health team members. OHT that performed planning actions (planning and programming activities based on local information; monitoring and analysis of oral health indicators; planning and programming together with the oral health team, and support for planning) had better results in care provision. As for the dental surgeon, it was found that complementary training in public health, better employment relationships and existence of a career plan were important for motivating the professional to provide care.
